# Correspondence to: hemophagocytic lymphohistiocytosis after SARS-CoV-2 vaccination

**DOI:** 10.1007/s15010-022-01820-z

**Published:** 2022-04-07

**Authors:** Rosanne Sprute, Marie-Lisa Hieber, Ron D. Jachimowicz

**Affiliations:** 1 Department I of Internal Medicine, Center for Integrated Oncology Aachen Bonn Cologne and Duesseldorf, Faculty of Medicine and University Hospital Cologne, University of Cologne, Cologne, Germany; 2 Cologne Excellence Cluster On Cellular Stress Response in Aging-Associated Diseases, University of Cologne, Cologne, Germany; 3 German Centre for Infection Research (DZIF), Partner Site Bonn-Cologne, Cologne, Germany; 4 Max-Planck-Institute for Biology of Ageing, Cologne, Germany; 5 Center for Molecular Medicine Cologne, University of Cologne, Cologne, Germany

Dear Sir,

We thank Mungmunpuntipantip and colleagues for their interest in our recently published case of a young adult female developing hemophagocytic lymphohistiocytosis (HLH) after BionTech mRNA SARS-CoV-2 vaccine [1].

In their correspondence, the authors ask about the patient’s immune and health status before vaccination. Indeed, many cases with HLH have a predisposing condition such as a genetic variant or an immunologic trigger, which may include malignancy or rheumatologic disorder [2]. In our patient, medical history was unremarkable for underlying diseases associated with HLH. The only pre-existing condition was a pilonidal disease which had been surgically drained 4 years ago. No other disease was identified during diagnostic workup. Sequencing of HLH associated genes was not performed in this case of adult HLH as genetic predisposition plays a major role in pediatric setting [3]. In addition, family history was unremarkable.

Mungmunpuntipantip et al. point out that the possibility of a concurrent medical problem should be discussed. We share the opinion that other differential diagnoses must be excluded when diagnosing HLH. The differential diagnosis of HLH includes several diseases characterized by cytopenia, fever, liver failure, and splenomegaly. In addition, HLH may develop in association with many of the conditions in its differential diagnosis [2].

In our case, important differential diagnoses have been ruled out by extensive diagnostics. In addition to imaging and microbiological, viral and rheumatological diagnostic approaches listed in the case report, blood cultures were repeatedly negative and serological testing for *Brucella* spp., *Chlamydia trachomatis*, *Coxiella burnetii*, *Echinococcus*, *Entamoeba histolytica*, *Leishmania*, *Treponema pallidum* and *Yersinia* was unremarkable. *Mycoplasma pneumoniae* infection has been excluded as trigger as follow-up titer did not show significant changes over time. We therefore consider the SARS-CoV-2 vaccine as the most plausible trigger for HLH in our patient. This observation is supported by other cases described in literature [1]. Regardless of the trigger, rapid recognition of the pathologic immune activation is critical to allow prompt treatment and exclusion of differential diagnoses should not delay therapy initiation.

In their correspondence, the authors also suggest that dengue infection must be considered as trigger for HLH. This is an interesting thought as HLH syndrome should be evaluated with the background of travel history and ethnicity. Our patient is of Turkish origin but was born and is living in Germany. She annually visits Turkey, the last time in summer 2021, 2 months prior to hospitalization. For this reason, we ruled out echinococcosis and leishmaniasis as triggers for HLH. Apart from that, travel history was unremarkable. Testing for dengue virus was not specifically performed because the patient’s medical and travel history did not suggest that the HLH syndrome was caused by dengue fever. In familial HLH, ethnicity is linked to a specific distribution of variants, which in turn are associated with phenotypic characteristics such as age of onset and disease course [4, 5].

Last but not least, we would like to emphasize, that the occurrence of HLH after vaccination is a very rare condition and the anecdotal reports to date suggest that any natural infection is a stronger trigger for HLH.
